# Molecular evolution of the H6 subtype influenza a viruses from poultry in eastern China from 2002 to 2010

**DOI:** 10.1186/1743-422X-8-470

**Published:** 2011-10-14

**Authors:** Guo Zhao, Xinlun Lu, Xiaobing Gu, Kunkun Zhao, Qingqing Song, Jinjin Pan, Quangang Xu, Zhiqiang Duan, Daxin Peng, Shunlin Hu, Xiaoquan Wang, Xiufan Liu

**Affiliations:** 1 College of Veterinary Medicine, Yangzhou University, Yangzhou, Jiangsu 225009, PR China

**Keywords:** H6 influenza viruses, poultry, phylogenetic analysis, molecular evolution

## Abstract

**Background:**

Although extensive data demonstrates that the majority of H6 duck isolates belonged to a single H6N2 virus lineage with a single gene constellation in southern China from 2000 to 2005, the prevalence of H6N2 virus in poultry in Eastern China is largely unknown.

**Results:**

Epidemiology revealed that H6N2 viruses were the most frequently detected influenza subtypes in live bird markets from 2002 to 2008 in Eastern China, but from 2009 onwards, they were replaced with novel H6N6 viruses. We phylogenetically and antigenically analyzed 42 H6 viruses isolated mainly in domestic ducks from 2002 to 2010 in Eastern China. Surprisingly, none of these isolates grouped with the previously described H6N2 viruses which belonged to a single H6N2 virus lineage with a single gene constellation in domestic ducks in southern China from 2000 to 2005. Two distinct hemagglutinin lineages were identified and they all underwent frequent reassortment with multiple virus subtypes from the natural gene pool, but few reassortants were persistent or prevalent.

**Conclusions:**

Five subtypes of H6 influenza viruses (H6N1, H6N2, H6N5, H6N6 and H6N8) cocirculated in Eastern China, which form a significant part of the natural influenza virus reservoir in domestic ducks, and significant viral reassortment is still ongoing in this species.

## Background

During the H5N1 outbreak in Hong Kong in 1997, A/Teal/Hong Kong/W312/97 (W312-like), an H6N1 avian influenza virus was isolated from a live bird markets (LBM). Subsequent characterisation of the virus revealed that seven gene segments were closely related to the A/Hong Kong/156/97 (156-like) influenza viruses, which infected 18 humans in 1997 [1,2]. In addition, A/Quail/Hong Kong/G1/97 (G1-like), an H9N2 virus, also shared the same six internal gene segments with the 156-like influenza viruses. In 1999, two individuals were infected with a G1-like influenza virus in Hong Kong [3,4]. The incident highlights the potential for avian influenza viruses to cross the species barrier and infect humans without adaptation in a mammalian host, such as the swine [5]; how these protein genes confer the ability of a virus to infect humans is not known, but influenza A viruses that possess these genes are of particular concern and pose a potential public health threat. The direction of gene flow among these three subtypes of influenza viruses (H5, H6, and H9) could not be determined, as they were all detected during the Hong Kong avian influenza incident [1]. In Taiwan, the H6N1 chicken isolates belonged to a unique lineage from 1972 to 2005, and this lineage of viruses differs from the H6N1 viruses circulating in Hong Kong and Southern China [6]. In southern China, the majority of H6N2 duck isolates belonged to a single H6N2 virus lineage with a single gene constellation from 2000 to 2005. Although accumulated information demonstrates that H6 viruses that are endemic in domestic ducks and terrestrial poultry (such as chickens or turkeys) seldom reassort [7-9], gene exchanges between viruses from domestic ducks and aquatic birds occur frequently [7]. In California, the H6N2 viruses were nonpathogenic in experimentally infected chickens [9], but pathogenicity tests showed that all the H6N1 viruses isolated in Taiwan were of low pathogenicity and might lead to economic loss when associated with other diseases [6].

In Eastern China, H6 influenza viruses were largely not reported. It is important to recognise that many mallards aggregate at favourable stopover or wintering sites in Eastern China, resulting in high local densities of this species. Such sites may be important for the transmission of influenza viruses between wild ducks and domestic ducks. In addition, the large mix of ducks, geese, chickens and swine in Eastern China creates an ideal environment for the generation of reassortant influenza viruses. To investigate the circulation and distribution of H6 influenza viruses in poultry in Eastern China and to determine whether H6 influenza viruses have been involved in the generation of the recent H5N1 and H9N2 variants, we engaged in extensive and systematic epidemiological surveillance of the LBM in ducks, geese and chickens from 2002 to 2010 and analysed the molecular evolution of the 42 H6 influenza viruses isolated mainly in domestic ducks, providing a glimpse of the genetic diversity of influenza viruses in poultry in Eastern China.

## Results

### H6 influenza viruses from poultry in Eastern China

**Figure 1:**
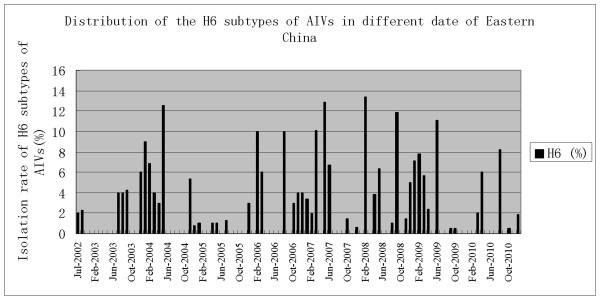
**Distribution of the H6 subtypes of AIVs in different date of Eastern China**.

Influenza virus surveillance of apparently healthy poultry species, including duck, goose and chicken, in LBM from July 2002 to December 2010 revealed that multiple influenza A virus subtypes were cocirculating in these birds, and H6 influenza viruses were present in aquatic and terrestrial poultry. A total of 375 H6 influenza viruses were isolated and identified from 13,103 samples (2,124 samples from chickens, 8,330 samples from ducks, 2,649 samples from geese). The isolation rate of the H6 viruses was 2.68%. Those viruses were prevalent year-round, but with a higher isolation rate during the winter (Figure 1). However, the isolation rate for each year varied markedly, from 0.63% to 4.38%. Of the poultry species, duck provided the main body of H6 isolates and had remarkably high isolation rates of 4.37%. In contrast, only eight H6 viruses was isolated from 2,124 chicken and three H6 viruses was isolated from 2,649 geese specimens collected during the same period and from the same market (Table 1).

**Table 1:** Isolation of H6 influenza viruses from terrestrial poultry in Eastern China during 2002 to 2010

Yr of isolation	No. (%) of H6-positive isolates	Total sample no.	No. of viruses sequenced
2002	15 (1.94)	773	5
2003	13 (1.40)	930	5
2004	43 (3.41)	1,261	2
2005	7 (0.63)	1,116	1
2006	39 (3.36)	1,160	0
2007	67 (3.89)	1,723	8
2008	89 (4.38)	2,031	5
2009	71 (3.29)	2,157	10
2010	31 (1.59)	1,952	6
Total	375(2.86)	13,103	42

### Phylogenetic analyses of H6 influenza viruses

**Figure 2:**
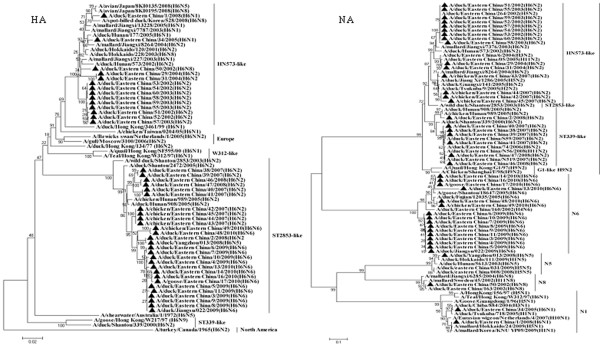
**Phylogenetic tree of the open reading frames of the HA and NA genes of the H6 influenza A viruses isolated in Eastern China**. Viruses highlighted with the black triangles are characterised in this study. The tree was constructed using the neighbour-joining method of MEGA 5.0, with 1,000 bootstrap trials to assign confidence to the groupings.

To understand the evolution of the H6 virus, 42 (22 H6N2, 16 H6N6, 2 H6N1, 1 H6N5 and 1 H6N8) of the 375 H6 isolates were sequenced. All the 42 H6 isolates sequenced were from ducks, except one H6N6 and four H6N2 from chickens and one H6N6 isolate from a goose. The nucleotide and amino acid similarity of the HA genes from 42 H6 isolates in this study were 84.5% to 99.9% and 87.1% to 99.8%, respectively. The phylogenetic trees (Figure 2) revealed that the 42 H6 virus isolates can be divided into two separate groups. The first H6 influenza group, represented by the A/wild duck/Shantou/2853/2003 (ST2853-like) virus, contained the H6N2, H6N5 and H6N6 viruses. This clade circulated in domestic ducks from 2007 to 2010. Sixty-seven percent of the H6 isolates sequenced clustered into this clade. Most importantly, all of the isolates from domestic ducks in this clade had the highest sequence similarity with the viruses isolated from geese and chickens during the same period and from the same markets, such as GS/EC/17/10 and Ck/EC/49/10. These findings suggest that the H6 influenza viruses have transmitted from ducks to geese and chickens in Eastern China. The second H6 influenza group, represented by the A/duck/Hunan/573/2002 (HN573-like) virus, was composed of the viruses isolated from poultry and wild birds. Viruses of this clade belonged to the Eurasian gene pool and included a diverse group of NA subtype combinations (N1, N2, N5 and N8), but none of these isolates grouped with the previously described H6N2 viruses of the A/duck/Shantou/339/2000 (ST339-like) and H6N1 viruses of the W312-like influenza subtype.

**Figure 3:**
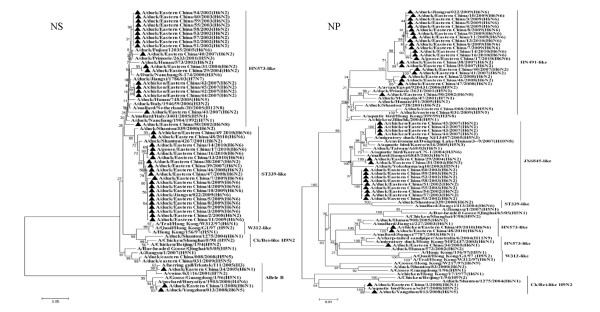
**Phylogenetic tree of the open reading frames of the NS and NP genes of the H6 influenza A viruses isolated in Eastern China**. Viruses highlighted with the black triangles are characterised in this study. The tree was constructed using the neighbour-joining method of MEGA 5.0, with 1,000 bootstrap trials to assign confidence to the groupings.

**Figure 4:**
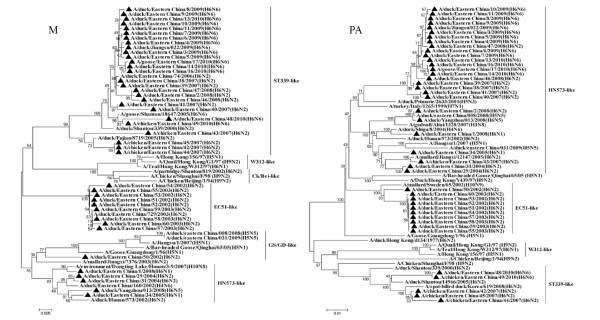
**Phylogenetic tree of the open reading frames of the M and PA genes of the H6 influenza A viruses isolated in Eastern China**. Viruses highlighted with the black triangles are characterised in this study. The tree was constructed using the neighbour-joining method of MEGA 5.0, with 1,000 bootstrap trials to assign confidence to the groupings.

**Figure 5:**
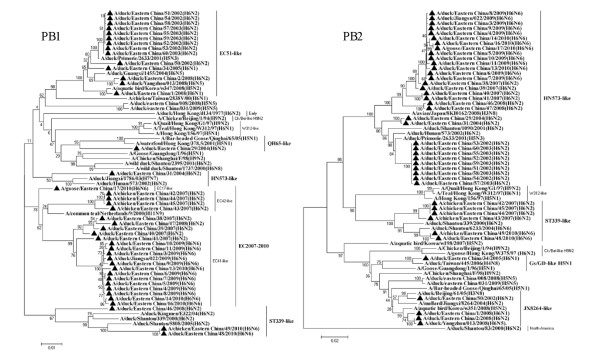
**Phylogenetic tree of the open reading frames of the PB1 and PB2 genes of the H6 influenza A viruses isolated in Eastern China**. Viruses highlighted with the black triangles are characterised in this study. The tree was constructed using the neighbour-joining method of MEGA 5.0, with 1,000 bootstrap trials to assign confidence to the groupings.

The NAs of all H6N1, H6N5, H6N6 and H6N8 isolates had no amino-acid deletions in the stalk region, similar to most of the H6N2 viruses, except for DC/EC/2/08, which had an 8-amino-acid deletion at the NA stalk region. Two N1 NAs of duck isolates had the highest sequence similarity with the viruses isolated from wild birds such as A/mallard/Hokkaido/24/2009 (H5N1), A/mallard/Korea/KNU/YP09/2009 (H1N1), A/duck/Chiba/884/2004 (H3N1) and A/duck/Tsukuba/718/2005 (H1N1). These findings suggest that reassortment has already occurred between the H6N1, H3N1, H1N1 and H5N1 virus lineages. Phylogenetic analysis of the N2 gene tree showed that it could be divided into two separate groups. The first influenza N2 subtype group, represented by the ST339-like influenza virus, confirmed a distinct lineage unique to domestic ducks. This clade was not associated with any wild aquatic bird, but multiple transmissions or gene mixing has already occurred between the ST339-like and ST2853-like influenza subtypes from 2007 to 2008. The second influenza N2 subtype group (HN573-like) was composed of the viruses isolated from poultry and wild birds and expressed different HA subtype combinations. The N5 isolate had a high similarity to two novel reassortant HPAI (H5N5) viruses (A/duck/eastern China/008/2008 and A/duck/eastern China/031/2009) [10]; the similarity between the subtypes was 95.2% for nucleotide sequence, and 97.3% to 97.5% for amino acid sequence, indicating that they evolved from the same parent or reassortment had already occurred between them. In addition, The N6 isolates may have derived from an aquatic ancestor, such as the A/duck/Eastern China/160/2002 (H4N6) influenza subtype. The N8 isolate had a high similarity to the A/mallard/Jiangxi/6285/2004 (H6N8) and A/mallard/Sweden/45/2002 (H11N8) influenza subtypes. These findings show the diversity of H6 influenza viruses in Eastern China. Phylogenetic analysis of the internal genes of influenza revealed that multiple transmissions or gene mixing has already occurred between migratory waterfowl, domestic aquatic birds and chickens (Figures 3, 4 and 5). Phylogenetic analysis of the polymerase acidic protein (PA) gene classified the H6 isolates into three major lineages. The HN573-like group had a similar lineage to the human isolate (A/Jiangsu/1/2007) and contained two novel reassortant HPAI (H5N5) viruses [10]; the high similarity between these viruses indicated that the H6 viruses isolated in Eastern China have donated genes for the generation of the H5 variants. The generalised phylogeny of polymerase basic protein 1 (PB1) genes revealed that that five influenza virus lineages cocirculated in Eurasia. The majority of the H6 viruses isolated from 2007-2010 were monophyletic (EC2007-2010) and formed a strong host restriction phylogenetic signal among domestic ducks, geese and chickens. In another branch, the H6N2 isolate (DC/EC/29/04) clustered with the A/Bar-headed Goose/Qinghai/65/05(QH65-like) virus, and the similarity between them was 94.4% for nucleotide sequence, and 98.4% for amino acid sequence, indicating that they evolved from the same parent. Thus, the molecular and phylogenetic results confirmed that H6 influenza viruses are undergoing frequent genetic recombination (Table 2), resulting in a large genomic pool that is shaped by selection in Eastern China.

**Table 2:** Gene composition of H6 subtype influenza viruses isolated from apparently healthy poultry in Eastern China*

Virus	Gene origin								
	
	Abbreviation	HA	NA	NS	NP	M	PB2	PB1	PA
A/duck/Eastern China/50/2002(H6N8)	Dk/EC/50/02	HN573-like	N8	HN573-like	HN491-like	GS/GD-like	JX8264-like	EC51-like	EC51-like
A/duck/Eastern China/51/2002(H6N2)	Dk/EC/51/02	HN573-like	HN573-like	HN573-like	JX6845-like	EC51-like	HN573-like	EC51-like	EC51-like
A/duck/Eastern China/52/2002(H6N2)	Dk/EC/52/02	HN573-like	HN573-like	HN573-like	JX6845-like	EC51-like	HN573-like	EC51-like	EC51-like
A/duck/Eastern China/53/2002(H6N2)	Dk/EC/53/02	HN573-like	HN573-like	HN573-like	JX6845-like	EC51-like	HN573-like	EC51-like	EC51-like
A/duck/Eastern China/54/2002(H6N2)	Dk/EC/54/02	HN573-like	HN573-like	HN573-like	JX6845-like	EC51-like	HN573-like	EC51-like	EC51-like
A/duck/Eastern China/55/2003(H6N2)	Dk/EC/55/03	HN573-like	HN573-like	HN573-like	JX6845-like	EC51-like	HN573-like	EC51-like	EC51-like
A/duck/Eastern China/57/2003(H6N2)	Dk/EC/57/03	HN573-like	HN573-like	HN573-like	JX6845-like	EC51-like	HN573-like	EC51-like	EC51-like
A/duck/Eastern China/58/2003(H6N2)	Dk/EC/58/03	HN573-like	HN573-like	HN573-like	JX6845-like	EC51-like	HN573-like	EC51-like	EC51-like
A/duck/Eastern China/59/2003(H6N2)	Dk/EC/59/03	HN573-like	HN573-like	HN573-like	JX6845-like	EC51-like	HN573-like	EC51-like	EC51-like
A/duck/Eastern China/60/2003(H6N2)	Dk/EC/60/03	HN573-like	HN573-like	HN573-like	JX6845-like	EC51-like	HN573-like	EC51-like	EC51-like
A/duck/Eastern China/29/2004(H6N2)	Dk/EC/29/04	HN573-like	HN573-like	HN573-like	JX6845-like	HN573-like	HN573-like	QH65-like	HN573-like
A/duck/Eastern China/31/2004(H6N2)	Dk/EC/31/04	HN573-like	HN573-like	HN573-like	JX6845-like	HN573-like	HN573-like	HN573-like	HN573-like
A/duck/Eastern China/34/2005(H6N1)	Dk/EC/34/05	HN573-like	N1	Allele B	HN573-like	HN573-like	GS/GD-like	EC51-like	HN573-like
A/duck/Eastern China/38/2007(H6N2)	Dk/EC/38/07	ST2853-like	ST339-like	ST339-like	HN491-like	ST339-like	HN573-like	EC41-like	HN573-like
A/duck/Eastern China/39/2007(H6N2)	Dk/EC/39/07	ST2853-like	ST339-like	ST339-like	HN491-like	ST339-like	HN573-like	EC41-like	HN573-like
A/duck/Eastern China/40/2007(H6N2)	Dk/EC/40/07	ST2853-like	ST339-like	HN573-like	HN491-like	ST339-like	HN573-like	EC41-like	HN573-like
A/duck/Eastern China/41/2007(H6N2)	Dk/EC/41/07	ST2853-like	ST339-like	HN573-like	HN491-like	ST339-like	HN573-like	EC41-like	HN573-like
A/chicken/Eastern China/42/2007(H6N2)	Ck/EC/42/07	ST2853-like	HN573-like	HN573-like	JX6845-like	ST339-like	ST339-like	EC42-like	ST339-like
A/chicken/Eastern China/43/2007(H6N2)	Ck/EC/43/07	ST2853-like	HN573-like	HN573-like	JX6845-like	ST339-like	ST339-like	EC42-like	HN573-like
A/chicken/Eastern China/44/2007(H6N2)	Ck/EC/44/07	ST2853-like	HN573-like	HN573-like	JX6845-like	ST339-like	ST339-like	EC42-like	ST339-like
A/chicken/Eastern China/45/2007(H6N2)	Ck/EC/45/07	ST2853-like	HN573-like	HN573-like	JX6845-like	ST339-like	ST339-like	EC42-like	ST339-like
A/duck/Eastern China/1/2008(H6N1)	Dk/EC/1/08	HN573-like	N1	Allele B	Ck/Bei-like	HN573-like	JX8264-like	EC51-like	HN573-like
A/duck/Eastern China/2/2008(H6N2)	Dk/EC/2/08	ST2853-like	ST339-like	ST339-like	HN491-like	ST339-like	JX8264-like	EC51-like	HN573-like
A/duck/Eastern China/46/2008(H6N2)	Dk/EC/46/08	ST2853-like	ST339-like	ST339-like	HN491-like	ST339-like	HN573-like	ST339-like	HN573-like
A/duck/Eastern China/47/2008(H6N2)	Dk/EC/47/08	ST2853-like	ST339-like	ST339-like	HN491-like	ST339-like	HN573-like	EC41-like	HN573-like
A/duck/Yangzhou/013/2008(H6N5)	Dk/YZ/013/08	ST2853-like	N5	Allele B	Ck/Bei-like	HN573-like	JX8264-like	EC51-like	HN573-like
A/duck/Jiangsu/022/2009(H6N6)	Dk/JS/022/09	ST2853-like	N6	ST339-like	HN491-like	ST339-like	HN573-like	EC41-like	HN573-like
A/duck/Eastern China/3/2009(H6N6)	Dk/EC/3/09	ST2853-like	N6	ST339-like	HN491-like	ST339-like	HN573-like	EC41-like	HN573-like
A/duck/Eastern China/4/2009(H6N6)	Dk/EC/4/09	ST2853-like	N6	ST339-like	HN491-like	ST339-like	HN573-like	EC41-like	HN573-like
A/duck/Eastern China/5/2009(H6N6)	Dk/EC/5/09	ST2853-like	N6	ST339-like	HN491-like	ST339-like	HN573-like	EC41-like	HN573-like
A/duck/Eastern China/6/2009(H6N6)	Dk/EC/6/09	ST2853-like	N6	ST339-like	HN491-like	ST339-like	HN573-like	EC41-like	HN573-like
A/duck/Eastern China/7/2009(H6N6)	Dk/EC/7/09	ST2853-like	N6	ST339-like	HN491-like	ST339-like	HN573-like	EC41-like	HN573-like
A/duck/Eastern China/8/2009(H6N6)	Dk/EC/8/09	ST2853-like	N6	ST339-like	HN491-like	ST339-like	HN573-like	EC41-like	HN573-like
A/duck/Eastern China/9/2009(H6N6)	Dk/EC/9/09	ST2853-like	N6	ST339-like	HN491-like	ST339-like	HN573-like	EC41-like	HN573-like
A/duck/Eastern China/10/2009(H6N6)	Dk/EC/10/09	ST2853-like	N6	ST339-like	HN491-like	ST339-like	HN573-like	EC41-like	HN573-like
A/duck/Eastern China/11/2009(H6N6)	Dk/EC/11/09	ST2853-like	N6	ST339-like	HN491-like	ST339-like	HN573-like	EC41-like	HN573-like
A/duck/Eastern China/13/2010(H6N6)	Dk/EC/13/10	ST2853-like	N6	ST339-like	HN491-like	ST339-like	HN573-like	EC41-like	HN573-like
A/duck/Eastern China/14/2010(H6N6)	Dk/EC/14/10	ST2853-like	N6	ST339-like	HN491-like	ST339-like	HN573-like	EC41-like	HN573-like
A/duck/Eastern China/16/2010(H6N6)	Dk/EC/16/10	ST2853-like	N6	ST339-like	HN491-like	ST339-like	HN573-like	EC41-like	HN573-like
A/goose/Eastern China/17/2010(H6N6)	GS/EC/17/10	ST2853-like	N6	ST339-like	HN491-like	ST339-like	HN573-like	EC17-like	HN573-like
A/chicken/Eastern China/49/2010(H6N6)	Ck/EC/49/10	ST2853-like	N6	ST339-like	HN573-like	ST339-like	ST339-like	ST339-like	ST339-like
A/duck/Eastern China/48/2010(H6N6)	Dk/EC/48/10	ST2853-like	N6	ST339-like	HN573-like	ST339-like	ST339-like	ST339-like	ST339-like

*Abbreviations: HN573-like, A/duck/Hunan/573/2002-like; ST2853-like, A/wild duck/Shantou/2853/2003-like; ST339-like, A/duck/Shantou/339/2000-like; JX6845-like, A/mallard/Jiangxi/6845/2003-like; HN491-like, A/duck/Hunan/491/2005-like; Gs/GD, goose/Guangdong/1/96-like; Ck/Bei-like, A/Chicken/Beijing/1/94-like; EC51-like, A/duck/Eastern China/51/2002-like; EC41-like, A/duck/Eastern China/41/2007-like; EC42-like, A/chicken/Eastern China/42/2007-like; EC17-like, A/goose/Eastern China/17/2010-like; QH65-like, A/Bar-headed Goose/Qinghai/65/05-like; and JX8264-like, A/mallard/Jiangxi/8264/2004-like.

### Molecular characterisation of H6 influenza viral genes

On the basis of this analysis, the HA gene of each H6 influenza virus isolated from poultry had an open reading frame of 1,701 bp that coded for 566 amino acid residues, with 16 amino acids coding a signal peptide. All H6 isolates possessed the sequence PQIETR↓G at the cleavage site (arrow) between HA1 and HA2, and no H6 isolates contained a sequence with multiple basic amino acids, which are found in highly pathogenic influenza A viruses [11]. Similar to H5 HA, the receptor-binding pocket of HA1 retains the amino acid residues Q224 and G226 (H6 numbering), which preferentially bind to the avian influenza virus receptor [12]. The consensus amino acid sequences revealed five potential N-linked glycosylation sites in HA1 (26 or 27, 39, 182, 306, and 311) and two in HA2 (498 and 557). The 29 strains isolated in 2007-2010 had N182T (20/29), N182R (8/29) and N182K (1/29) mutations, and the N306K substitution in DC/EC/10/09 led to the loss of potential glycosylation sites in the HA protein. The analysis of the NA sequences showed that there was no mutation at the H274Y position of the NA protein (NA of GS/GD number), indicating the isolates maybe sensitive to neuraminidase inhibitor drugs such as oseltamivir phosphate [13,14].

The analysis of internal gene amino acid sequences showed that the K317 and I198 positions of the PB1 protein, which are associated with pathogenicity in mice, were mutated to M198 (40/42), T198 (1/42), V198 (1/42) and K317 (1/42), respectively [15]. The 627 amino acid residue of PB2 is considered to be the predominant factor for the host range and the replication of the influenza A virus [16], and the PB2 amino acid residue at position 627 of the H6 isolates was E rather than K. The PB2 position 701, which is considered to be the molecular marker of cross-species transmission from duck to human in the H5N1 duck influenza A virus [17], was a D in the 42 isolates. The PB2 amino acid residues E and D at the 627 and 701 positions, respectively, were characteristic of the avian influenza virus. Studies have shown that the virulence of the influenza virus for humans is relative to the viral resistance of the antiviral effects of cytokines, such as IFN, and the mutation of D92E in the NS1 protein promotes a greater resistance to these cytokines [18]. However, the 42 isolates in this study did not have mutations at position 92 in the NS1 protein. In addition, no virus had an N31 substitution in the M2 protein, which is associated with amantadine resistance [13].

### Antigenic analysis

To understand the antigenic properties of HN573-like and ST2853-like H6 viruses, representatives from each of the two H6 lineages were tested by hemagglutinin inhibition test (HI) using a panel of reference antisera (Table 3). None of the HN573-like viruses reacted well with the ST2853-like H6 viruses.

**Table 3:** Antigenic analysis of the H6 subtype of influenza viruses isolated from apparently healthy poultry in Eastern China

Viruses	Group	Titre to antiserum:							
	
		Dk/EC/51/02	Dk/EC/60/03	Dk/EC/31/04	Dk/EC/34/05	Dk/EC/38/07	Dk/EC/2/08	Dk/EC/8/09	Dk/EC/13/10
Dk/EC/50/02(H6N8)	HN573-like	2,048	2,048	2,048	2,048	256	128	64	128
Dk/EC/51/02(H6N2)	HN573-like	2,048	1,024	1,024	2,048	128	32	32	64
Dk/EC/52/02(H6N2)	HN573-like	2,048	2,048	1,024	2,048	512	64	64	256
Dk/EC/53/02(H6N2)	HN573-like	2,048	2,048	2,048	2,048	512	128	128	512
Dk/EC/54/02(H6N2)	HN573-like	2,048	2,048	2,048	2,048	512	128	128	128
Dk/EC/55/03(H6N2)	HN573-like	2,048	1,024	1,024	2,048	256	128	64	256
Dk/EC/57/03(H6N2)	HN573-like	2,048	2,048	2,048	2,048	1,024	128	128	256
Dk/EC/58/03(H6N2)	HN573-like	2,048	2,048	1,024	2,048	1,024	128	128	512
Dk/EC/59/03(H6N2)	HN573-like	2,048	2,048	2,048	2,048	512	64	64	512
Dk/EC/60/03(H6N2)	HN573-like	2,048	1,024	2,048	2,048	256	64	64	256
Dk/EC/29/04(H6N2)	HN573-like	2,048	1,024	2,048	2,048	1,024	512	128	512
Dk/EC/31/04(H6N2)	HN573-like	2,048	1,024	2,048	2,048	512	256	64	1,024
Dk/EC/34/05(H6N1)	HN573-like	2,048	1,024	1,024	2,048	512	256	64	256
Dk/EC/1/08(H6N1)	HN573-like	2,048	1,024	512	1,024	512	128	128	256
Dk/EC/38/07(H6N2)	ST2853-like	256	64	256	512	1,024	512	512	1,024
Dk/EC/39/07(H6N2)	ST2853-like	1,024	512	1,024	1,024	1,024	1,024	512	2,048
Dk/EC/40/07(H6N2)	ST2853-like	512	512	1,024	1,024	1,024	1,024	512	2,048
Dk/EC/41/07(H6N2)	ST2853-like	1,024	256	1,024	1,024	512	1,024	256	2,048
Ck/EC/42/07(H6N2)	ST2853-like	512	512	1,024	1,024	1,024	1,024	512	2,048
Ck/EC/43/07(H6N2)	ST2853-like	512	1,024	1,024	1,024	1,024	1,024	1,024	2,048
Ck/EC/44/07(H6N2)	ST2853-like	512	512	256	1,024	1,024	1,024	128	2,048
Ck/EC/45/07(H6N2)	ST2853-like	512	512	1,024	1,024	1,024	1,024	256	2,048
Dk/EC/2/08(H6N2)	ST2853-like	1,024	128	64	64	256	1,024	256	1,024
Dk/EC/46/08(H6N2)	ST2853-like	1,024	128	256	512	256	1,024	256	2,048
Dk/EC/47/08(H6N2)	ST2853-like	1,024	128	512	1,024	1,024	1,024	512	2,048
Dk/YZ/013/08(H6N5)	ST2853-like	512	256	512	512	256	512	256	512
Dk/JS/022/09(H6N6)	ST2853-like	512	128	256	256	128	512	2,048	1,024
Dk/EC/3/09(H6N6)	ST2853-like	1,024	512	256	1,024	512	1,024	2,048	2,048
Dk/EC/4/09(H6N6)	ST2853-like	128	64	256	512	128	256	2,048	2,048
Dk/EC/5/09(H6N6)	ST2853-like	512	256	512	512	256	512	2,048	1,024
Dk/EC/6/09(H6N6)	ST2853-like	256	128	256	512	512	256	2,048	2,048
Dk/EC/7/09(H6N6)	ST2853-like	256	128	128	128	512	1,024	2,048	1,024
Dk/EC/8/09(H6N6)	ST2853-like	256	512	256	128	256	256	2,048	2,048
Dk/EC/9/09(H6N6)	ST2853-like	512	512	256	512	128	512	2,048	2,048
Dk/EC/10/09(H6N6)	ST2853-like	512	128	256	512	256	1,024	2,048	2,048
Dk/EC/11/09(H6N6)	ST2853-like	512	128	512	256	128	1,024	2,048	2,048
Dk/EC/13/10(H6N6)	ST2853-like	1,024	256	512	1,024	1,024	1,024	256	2,048
Dk/EC/14/10(H6N6)	ST2853-like	1,024	256	512	1,024	512	1,024	256	2,048
Dk/EC/16/10(H6N6)	ST2853-like	256	256	512	512	512	1,024	512	2,048
GS/EC/17/10(H6N6)	ST2853-like	512	128	256	512	256	512	256	2,048
Ck/EC/49/10(H6N6)	ST2853-like	256	64	128	256	512	1,024	128	2,048
Dk/EC/48/10(H6N6)	ST2853-like	512	64	256	512	512	1,024	512	2,048

## Discussion

H6 subtypes of influenza viruses are the most abundantly detected influenza subtype in wild birds and poultry, and they have a broader host range than any other subtype [19]. However, in Eastern China, the H6 subtypes are largely not reported. The results of the present study indicate that at least five subtypes of influenza viruses, including H6N1, H6N2, H6N5, H6N6 and H6N8 subtypes, cocirculated in this region. H6N2 viruses were the most frequently detected influenza subtypes in domestic ducks from 2002 to 2008, but from 2009 onwards, they were replaced with novel H6N6 viruses. Previous studies have shown that ducks and shorebirds are the natural reservoirs of influenza A viruses [20,21]. However, the role of domestic ducks in influenza virus ecology has not been fully defined. In this study, phylogenetic analysis of viruses isolated in domestic duck revealed that these viruses cannot be distinguished from the viruses detected directly from migratory birds. This suggests that domestic ducks in Eastern China form a significant part of the natural influenza virus reservoir. Even though interspecies transmissions of the H6 subtype virus from domestic ducks to terrestrial poultry are not common [7], our findings suggest that the H6 influenza A virus has transmitted in geese and chickens from ducks in Eastern China. Y. Guan has analysed 170 H6 viruses isolated from domestic ducks from 2000 to 2005 in southern China and found that three distinct hemagglutinin lineages were identified. Group I (ST339-like influenza viruses) contained the majority of isolates with a single internal gene complex in domestic ducks. Group II (ST2853-like influenza viruses) was derived from reassortment events in which the surface genes of group I viruses were replaced with novel surface genes. Group III (HN573-like influenza viruses) undergo frequent reassortment with multiple virus subtypes from the natural gene pool [7]. It is important to realize that many mallards and waterfowl move simply from eastern China to southern China by migration or live poultry trade. Our findings further validate that domestic ducks in China mediate the interaction of viruses between different gene pools and facilitate the generation of novel influenza virus variants circulating in poultry, but genotypic analyses demonstrated that the H6 viruses tested were different from the ST339-like [7] and W312-like viruses [8] circulating in southern China, and these viruses have undergone extensive reassortment.

In California, H6N2 viruses have been commonly isolated from chickens with clinical signs of infection [9]. A drop in egg production and mild respiratory distress were observed in chickens infected with the H6N1 viruses isolated in Taiwan [6]. In contrast, the Eastern China H6 influenza viruses were mostly isolated from ducks, geese and chickens that showed no clinically significant signs of disease. Even though gene exchange between the established H6 virus lineage and the natural gene pool occurred sporadically throughout the surveillance period, few reassortants were persistent or prevalent, which might result from that these reassortant viruses did not have significant fitness over the viruses with established internal gene complexes. Moreover, the H6 viruses have been found to be of contribution to the genetic diversity of H5 and H9N2 viruses.

## Conclusions

In conclusion, five subtypes of H6 influenza viruses (H6N1, H6N2, H6N5, H6N6 and H6N8) cocirculated in Eastern China, which form a sporadic part of the natural influenza virus reservoir in this region, and significant viral reassortment is still ongoing in this species. Despite the relatively intense surveillance studies that have been performed for 10 years in Eastern China, our understanding of the epidemiology of H6 subtype avian influenza viruses in poultry is still limited. Surveys covering a wider region may provide more insight into the year-round perpetuation of influenza viruses in poultry and wild birds.

## Materials and methods

### Virus isolation and identification

A total of 13,103 cloacal swab samples were collected randomly from apparently healthy poultry species, including duck, goose and chicken, monthly in LBM in Yangzhou, Jiangsu Province, China from July 2002 to December 2010. Cloacal swabs were collected from each consignment shipped to the LBM from local farms or were introduced from neighbouring provinces in Eastern China such as Shandong, Anhui, Zhejiang, and Shanghai. Cloacal swabs were maintained in transport medium containing antibiotics and kept at 4°C until transported to the laboratory. Viruses were inoculated into embryonated chicken eggs, and the presence of virus in the allantoic fluids of the embryos were determined by a rapid plate hemagglutination test using 0.5% suspensions of chicken erythrocytes. The HA subtype and the NA subtype were identified using reverse transcription-polymerase chain reaction (RT-PCR) reported by Lee [22] and Qiu [23] and were confirmed by nucleotide sequencing.

### Viral sequencing

Viral RNA was extracted from infected allantoic fluids with the Trizol LS reagent (Invitrogen, Carlsbad, CA). Viral RNA was reverse transcribed with the 12 bp primer 5'-AGCAAAAGCAGG-3'. PCR was performed using specific primers as described by Hoffmann et al. [24]. PCR products were purified with the TaKaRa Agarose Gel DNA Purification Kit Ver. 2.0 (TaKaRa, Dalian, China) and sequenced by the Nanjing GenScript Biotech Co., Ltd. The H6 influenza viruses sequenced and their abbreviations used in this study are listed in Table 2.

### Phylogenetic analysis and evolutionary analysis

The published influenza viruses sequences used for phylogenetic comparison in this study were obtained from the Influenza Sequences Database (http://www.flu.lanl.gov). Editing and analysis of sequence data was performed with BioEdit 7.0, and alignment of sequence data was performed with Clustal X. Phylogenetic trees based on coding sequences of individual genes were constructed using the Kimura two-parameter model and the neighbour-joining algorithm using the MEGA program (version 5.0) with 1,000 bootstraps. All of the branches supported by >50% bootstrap values were considered to be in the same group in the trees.

### Nucleotide sequence accession numbers

The sequence data obtained in this study are available in GenBank under the accession numbers JF965018-JF965337, GU220596-GU220603 and GU324771-GU324778.

### Antigenic analysis

The antigenic characteristics of the representative H6 influenza viruses were compared by a hemagglutination inhibition (HI) assay with post-infection chicken antisera raised against Dk/EC/51/02 (H6N2), Dk/EC/60/03 (H6N2), Dk/EC/31/04(H6N2), Dk/EC/34/05 (H6N1), Dk/EC/38/07(H6N2), Dk/EC/2/08 (H6N2), Dk/EC/8/09(H6N6), and Dk/EC/13/10(H6N6) viruses that were generated in our laboratory.

## Competing interests

The authors declare that they have no competing interests.

## Authors' contributions

GZ carried out the study design, participated in the sequence alignment and drafted the manuscript. XL, XG, KZ, QS, JP, QX and ZD participated in sample collection and virus isolation from poultry in Eastern China. DP, SH and XW contributed to the design of the study and revision of the manuscript. XL conceived of the study, provided consultation and coordination, and helped to draft the manuscript. All authors read and approved the final manuscript.
